# Bioactive Peptides and Evaluation of Cardiac Cytoprotective Effects of Red Millet Yellow Wine as Functional Food

**DOI:** 10.3390/foods13244111

**Published:** 2024-12-19

**Authors:** Zehui He, Yu Zhou, Shuang Li, Wen Li, Yingxin Zhang, Cancan Guo, Zexin Guo, Bo Wei, Yuefeng Bi

**Affiliations:** School of Pharmaceutic Sciences, Zhengzhou University, Zhengzhou 450001, China; 18817773761@163.com (Z.H.); zhouyu20001122@163.com (Y.Z.); lis0801@163.com (S.L.);

**Keywords:** red millet yellow wine, peptide extraction, isolation and identification, cardioprotective effect, oxidative stress, Sirt1/Nrf2 pathway

## Abstract

Red millet yellow wine, a functional beverage fermented from grain, has physiological functions including relieving cardiovascular diseases. However, the active components and mechanism of red millet yellow wine largely remain to be elucidated. In this study, bioactive peptides in red millet yellow wine and the cardiac cytoprotective effects were first investigated. A single-factor test and response surface method were used to optimize the solvent precipitation method to purify bioactive peptides. The final peptide content was up to 72.23%. Analysis of liquid chromatography–tandem mass spectrometry indicated a high antioxidative potential of the identified peptides. Multiple activity assays in vitro revealed that red millet yellow wine (1 mg/mL), particularly peptides (0.1 mg/mL), could protect H9c2 cells from H_2_O_2_-induced oxidative damage, thereby improving cell viability. At the mechanistic level, the antioxidant effect of bioactive peptides was achieved through strengthening antioxidative stress capacity and attributed to the activation of the Sirt1/Nrf2 pathway, indicating that peptides may be the main active components responsible for the cardiac cytoprotective effects of red millet yellow wine. These results are expected to provide a reference for further exploration of the health benefits of red millet yellow wine.

## 1. Introduction

Chinese yellow wine is a traditional fermented grain wine with a history spanning over 2500 years [1]. Depending on the difference in the region and primary cereal materials, millet in North China and sticky rice in South China, Chinese yellow wine can be classified into the following two categories: North China yellow wine and South China yellow wine [2].

Red millet yellow wine, a quintessential northern yellow wine, is fermented from red millet, a characteristic crop with high crude protein content and abundant amino acids of Nanyang City, Henan Province [3]. When red millet is broken down with lactic acid bacteria, fungi, and yeast, a wealth of bioactive constituents, including proteins, peptides, GABA (γ-aminobutyric acid), amino acids, vitamins, and polyphenols, are produced, resulting in pharmacological and nutritional benefits of yellow wine [4,5]. The traditional belief associated with red millet rice wine is that it possesses the potential to enhance blood circulation and foster cardiovascular well-being by mobilizing and dredging the meridians. Epidemiological and clinical studies have pointed out that drinking light to moderate amounts of wine is associated with decreased incidence of cardiovascular disease [6,7]. These beneficial effects may be related to the antioxidant and anti-inflammatory effects of melatonin, polysaccharides, polypeptides, or glutathione [8,9].

It has been demonstrated that yellow wine contains the highest nitrogen content of all brewed wines, wherein these compounds consist mainly of peptides and amino acids [10]. Polypeptides present in yellow wine exhibit good ABTS (2, 2′-azino-bis (3- ethylbenzothiazoline-6-sulphonic acid)) and DPPH (2, 2-diphenyl-1-picrylhydrazyl) radical scavenging activities and antioxidant activities [11,12]. In our previous study, red millet yellow wine and glutinous yellow wine were studied to identify their potential cardioprotective effects [13]. Interestingly, Xinye red millet yellow wine, which is rich in peptides (15.6 g/L), was found to exhibit the strongest cardioprotective effect, indicating that the peptides may play an irreplaceable role in the myocardial protection of yellow wine. Nevertheless, due to challenges associated with peptide extraction, characterization, and identification from intricate matrices, there are few research studies, particularly biological activity studies, on polypeptides in yellow wine [14].

Given the dominant role of oxidative stress in cardiac injury caused by myocardial ischemia or myocardial infarction, there is a requirement to discover the potential cardioprotective effect and active components of red millet yellow wine [15]. In the research, the purification technology of active peptides was studied with the solvent precipitation method. Cardioprotective effects of red millet yellow wine and peptides were systematically researched using an H_2_O_2_-induced oxidative stress model. Finally, a preliminary investigation is conducted into cardioprotective mechanisms to provide an important theoretical basis for the functional development and utilization of red millet yellow wine.

## 2. Materials and Methods

### 2.1. Single-Factor Design for Peptide Extraction

Red millet yellow wine (500 mL) was concentrated at 55 °C under reduced pressure, then dried to a constant weight to determine the weight of the total extract of the red millet yellow wine (RYW-TE), which was sealed for later use. Subsequently, a sample (1 g) of the RYW-TE was weighed and then dispersed in ethanol (1 mL) under ultrasonication conditions for 10 min. After this time, acetone (3 mL) was added to the dispersion to generate a flocculent precipitate, which was subjected to centrifugation to obtain a precipitate. This procedure was repeated with the retained RYW-TE until the extracted supernatant gave a negative result upon testing with the Folin and Ciocalteu phenol reagent, indicating that all proteins had been recovered in precipitation. The obtained precipitate was denoted red millet yellow wine–total peptide (RYW-TP). A single-factor experimental design was established to determine the optimal conditions for peptide extraction, wherein a number of variables were investigated, as listed in Table 1.

### 2.2. Optimization of RYW-TP

The peptide extraction process was adequately optimized using the Box–Behnken response surface method and the results obtained for the above single-factor experiments [16]. For this purpose, three independent variables were investigated at three levels (−1, 0, and 1): (A) the acetone/ethanol ratio, (B) the solid/liquid ratio, and (C) the centrifugal speed. The coding and level of experimental design factors are shown in Table 2. The peptide content of the RYW-TP specimen was used as the response variable. Design Expert 8.0.6 software was employed to design the experimental scheme [16].

### 2.3. Molecular Weight (Mw) Distribution

The activity of a functional peptide depends heavily on the molecular weight distribution of its peptides [17]. With this in mind, the Mw distribution in the RYW-TP specimen was evaluated according to the method of Liu et al. [18]. More specifically, RYW-TP (100 mg) was dissolved in the mobile phase to obtain a sample solution (10 mg/mL), which was subsequently filtered through a 0.45 μm microporous filter membrane and injected into the HPLC instrument (10 μL sample volume). Analysis of the sample solution was carried out by high-performance liquid chromatography (HPLC, 220 nm detection; Waters 2695 HPLC System, Waters, Milford, MA, USA) using a TSKgel 2000SWXL column (300 mm × 7.8 mm, 30 °C column temperature). The mobile phase consisted of acetonitrile/water/trifluoroacetic acid, 40:59.9:0.1 (*v*/*v*/*v*), at a flow rate of 0.5 mL/min. Standard solutions of cytochrome C (12,384 Da), aprotinin (6500 Da), bacitracin (1422 Da), glycine–glycine–tyrosine–arginine (451 Da), and glycine–glycine–glycine (189 Da) were prepared as 0.2 mg/mL solutions and filtered through a 0.45 μm microporous filter membrane prior to analysis.

### 2.4. Amino Acid Analysis of the RYW-TP

The amino acid compositions of the peptides were estimated according to the acid hydrolysis method using an automatic amino acid analyzer (Biochrom30+, Biochrom, Cambridge, UK). Initially, the desired sample (100 mg) was hydrolyzed using HCl solution (6 M, 10 mL) in a sealed hydrolysis tube filled with N_2_ at 110 °C for 22–24 h. After this time, the mixture solution was cooled to 20 °C and filtered through a 0.45 μm filter membrane into a 50 mL volumetric flask. Water was added to the scale line. Subsequently, an aliquot (2 mL) of this sample was evaporated at 45 °C for deacidification and drying until a small amount of solid residue remained in the flask. Finally, this solid residue was dissolved in the sodium citrate buffer solution (pH = 2.2, 2 mL) and filtered through a 0.45 μm microporous filter membrane prior to analysis.

### 2.5. Identification of the RYW-TP

Identification of the peptides present in RYW-TP was conducted by means of liquid chromatography–mass spectrometry (LC-MS; Nano-LC-Q-Orbitrap-MS/MS; Thermo EASY nLC and Thermo Scientific Q Exactive™ MS, Thermo Fisher Scientific, Waltham, MA, USA). The samples were separated using an Acclaim PepMap RSLC C18 reversed-phase column (75 μm × 150 mm). Mobile phase A consisted of a 0.1 vol% solution of formic acid in water, while mobile phase B consisted of a 0.1 vol% formic acid solution in a mixture of acetonitrile (80%) and water (20%). The elution method was as follows: 0–3 min, 97% A; 3–4.5 min, 97–92% A; 4.5–28 min, 92–68% A; 28–31 min, 68–56% A; 31–34 min, 56–1% A; 34–30 min, 1% A. The injection volume was 8 μL and the flow rate was 500 nL/min. Thermo Xcalibur software 4.1.31.9 was used to collect the mass spectral data in the positive ion mode. The primary mass resolution was 120,000, the *m*/*z* mass range was 350–1550, the auto gain control (AGC) was set to 4 × 10^5^, and the maximum ion implantation time was 50 ms [19]. The secondary mass resolution was 50,000, the Full MS/dd-MS2 was set to 20, the normalized collision energy (NCE) was set to 32%, the AGC was set to 1× 10^5^, and the maximum ion implantation time was 100 ms. The amino acid sequences of the peptides were obtained using de novo sequencing and Peaks Studio 8.0 software with searching of the UniProt protein database [20]. 

### 2.6. Evaluation of the Cardioprotective Effect of Red Millet Yellow Wine Against H_2_O_2_-Induced Oxidative Stress

#### 2.6.1. Cell Culture and Viability Assays

H9c2 cells were cultured as described by Zhang et al. with slight modifications [21]. More specifically, the H9c2 cells were cultivated at 37 °C in a humidified environment containing 5% CO_2_ and using DMEM supplemented with 10% bovine serum. The cell concentration was adjusted to 5 × 10^3^ cells/well in the growth medium, and the cell suspension was seeded into 96-well microstate plates and allowed to grow. Subsequently, the cells were allowed to grow and adhere to the wells for a 48 h period prior to exposure to different concentrations of the RYW-TE and RYW-TP sample solutions (0.1, 0.5, 1, 2.5 mg/mL) for 24 h for cytotoxicity evaluations. The protective effects of these samples on the H9c2 cells were determined by establishing an H_2_O_2_-induced (300 μM) oxidative stress model. For this purpose, five experimental groups were employed, including the blank group (control group), the damage group (300 μL of H_2_O_2_ solution), the contrast group (H_2_O_2_ + 10 μM resveratrol antioxidant), the RYW-TE-protected group (H_2_O_2_ + 0.5, 1, 2 mg/mL RYW-TE), and the RYW-TP-protected group (H_2_O_2_ + 0.05, 0.1, 0.2 mg/mL RYW-TP). The cell viability was determined via a colorimetric approach at 450 nm using the standard Cell Counting Kit-8 (CCK-8) in a 96-well plate. All measurements were repeated three times. The effects of the RYW-TE and RYW-TP specimens on the cell viability were also observed.

#### 2.6.2. Determination of the Malondialdehyde Content

The malondialdehyde (MDA) content was measured in the supernatant of the lysed cells using a commercial test kit (Jiancheng Biochemical Inc., Nanjing, China), wherein MDA reacts with the thiobarbituric acid (TBA) present in the test kit to form a red conjugate with a maximum absorbance wavelength of 530 nm. The extent of lipid peroxidation was quantified by estimating the MDA concentration, since this is the most abundant aldehyde resulting from lipid peroxidation.

#### 2.6.3. Determination of the Superoxide Dismutase Levels

A superoxide dismutase (SOD) assay kit (Jiancheng Biochemical Inc., Nanjing, China) was used to spectrophotometrically assess the superoxide scavenging activities of the RYW-TE and RYW-TP specimens at 450 nm. This test was based on the reaction between the superoxide radicals (from the xanthine/xanthine oxidase, XOD, system) and the Nitro Blue Tetrazolium chloride (NBT) reagent to generate formazan dye, whose concentration was determined based on the adsorption of the sample at 450 nm.

#### 2.6.4. Determination of the Glutathione Content

The content of glutathione (GSH), which is the most important sulfhydryl antioxidant in cells, was estimated using a GSH colorimetric assay kit (Jiancheng Biochemical Inc., Nanjing, China). In the kit, 5, 5′-dithio-bis(2-nitrobenzoic acid) (DTNB) and GSH reacted to form a yellow complex with a distinctive absorption peak at 405 nm. The absorbance at this wavelength was proportional to the GSH content in the solution.

#### 2.6.5. Lactic Acid Dehydrogenase Release Assay

The lactic acid dehydrogenase (LDH) levels were also used to assess the cytotoxicities of the RYW-TE and RYW-TP specimens. The test solution was prepared according to the instructions of the LDH Cytotoxicity Assay kit. Cultured H9c2 cells were seeded into 96-well cell culture plates, aspirated from the media, and washed once with PBS solution. After changing to a fresh culture medium, the wells were divided into the following four groups: cell-free culture wells (blank control wells), untreated control cells (control wells), untreated cells for subsequent lysis (maximum enzyme activity control wells), and drug-treated cells (drug-treated wells). Appropriate amounts of RYW-TP and RYW-TE were added and the regular culture was continued. At a time of 1 h prior to the predetermined time point, the cell culture plate was removed from the cell incubator and the LDH release reagent included in the kit was added to the control well of the sample (volume = 10% of the original culture volume). After adding the LDH release reagent, the medium was repeatedly mixed before continuing incubation in the cell incubator. After reaching the predetermined time, the cell culture plates were centrifuged using a multi-well plate centrifuge (400× *g*) for 5 min. An aliquot (120 μL) of supernatant from each well was added to the corresponding wells of a new 96-well plate and the absorbance of the solution was measured at 490 nm.

#### 2.6.6. Reactive Oxygen Species Generation

The reactive oxygen species (ROS) generated were detected by a fluorescent probe, namely 2′, 7′-dichlorofluorescin diacetate (DCFH-DA), which was provided in the ROS assay kit (Beyotime Biotechnology Co., Ltd., Shanghai, China). DCFH-DA itself exhibits no fluorescence and can enter the cells freely by penetrating the cell membrane. DCFH-DA is then hydrolyzed by the cellular esterases to produce dichlorodihydrofluorescein (DCFH), which is then oxidized by the ROS to produce the fluorescent 2′, 7′-dichlorofluorescein (DCF), whose fluorescence intensity can be used to quantify the ROS levels. The fluorescence molecular probe was prepared using a DCFH-DA/serum-free medium ratio of 1:1000 and was stored at 4 °C in the absence of light. After the preparation of each group of cells, the culture medium was removed, the cells were washed twice with PBS, the prepared probe was added to the wells, and the wells were mixed evenly and covered. Subsequently, the resulting mixtures were incubated at 37 °C for 20 min, washed with the serum-free DMEM culture solution (3×), and subjected to semi-quantitative fluorescence analysis using ImageJ software 1.53t [22].

#### 2.6.7. Western Blotting

The degrees of protein expression and phosphorylation were determined using Western blotting [23]. For this purpose, the total soluble proteins were extracted from cultured H9c2 cells using RIPA lysis buffer containing the protease inhibitor phenylmethylsulfonyl fluoride (PMSF). The proteins were quantified to obtain the same amount of proteins according to the BCA protein quantification kit (Sevierbio Co., Ltd., Wuhan, China). More specifically, the protein sample and 5 loading buffers were prepared in a 4:1 ratio, mixed with vortex oscillation, denatured in boiling water for 5 min, cooled to 25 °C, subjected to centrifugation at 3500 rpm, and stored at −20 °C for later use. Equal amounts of protein were separated by sodium dodecyl sulfate–polyacrylamide gel electrophoresis (SDS-PAGE) and transferred to PVDF membranes. Subsequently, the appropriate primary antibodies were added to the PVDF membranes as follows, and incubated overnight at 4 °C: silent information regulator 1 (Sirt1, 1:1000, 50 μg), Nrf2 (1:500, 50 μg), NAD (P)H dehydrogenase quinone 1 (NQO1, 1:1000, 50 μg), and heme oxygenase 1 (HO-1, 1:1000, 50 μg). The PVDF membranes were then washed five times with PBS containing Tween-20 (PBST) for 5 min each to remove the primary antibodies. After diluting the secondary antibody with a 1% solution of skimmed milk in water, it was incubated at 20 °C for 2 h and washed with PBST (5×). The PVDF membranes were then placed in a clean PBST solution, and the protein bands were visualized using an ECL kit.

### 2.7. Statistical Analysis

All experiments were conducted at least in triplicate. All experimental data were analyzed using GraphPad Prism 9.0 and SPSS 25.0 software. A one-way analysis of variance (one-way ANOVA) with a Tukey post hoc test was used for multiple group comparisons. *p* < 0.05 indicates a statistical difference in the experimental data [24].

## 3. Results and Discussion

### 3.1. Single-Factor Experiments

**Figure 1:**
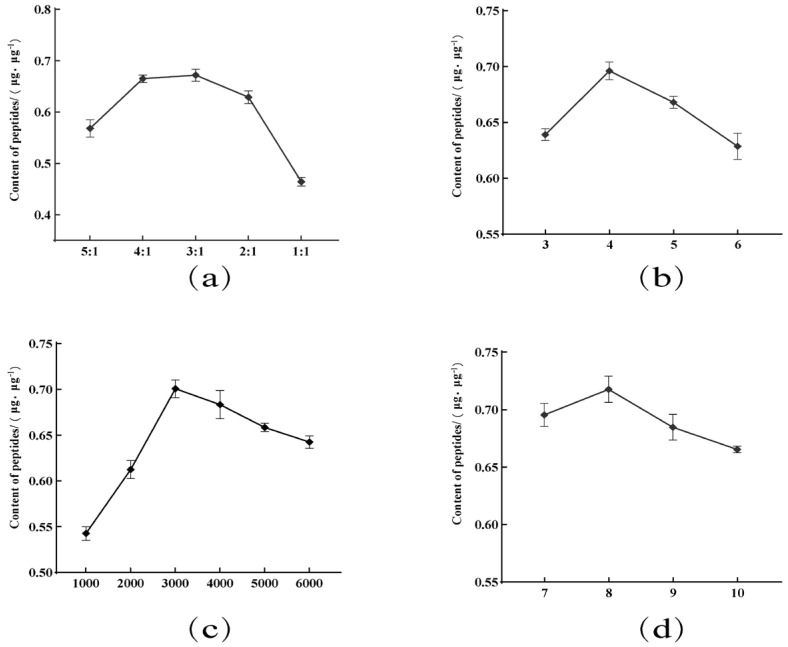
Effects of different extraction conditions on the peptide content of the RYWTP extract: (**a**) the acetone/ethanol ratio, (**b**) the solid/liquid ratio, (**c**) the centrifugal speed (rmp), and (**d**) the number of extractions (times).

The acetone/ethanol ratio is a key variable since it influences the solvent polarity and affects the peptide extraction yield. As shown in Figure 1a, upon increasing the acetone/ethanol ratio from 5:1 to 3:1, the peptide extraction yield increased and peaked at 67.20%. This was attributed to the fact that, as the solvent polarity increases (i.e., upon increasing the ethanol proportion), the polarity of the extraction solution also increases, and substances with lower polarities than the active peptides are also extracted from the supernatant. However, as the ratio was further increased from 3:1 to 1:1, all peptide peaks declined considerably in intensity. This was likely due to the solution polarity being too high, leading to the dissolution of some hydrophilic peptides.

In addition, as shown in Figure 1b, the solid/liquid ratio had a significant effect on the content of active peptides extracted, until reaching a ratio of 4:1. Beyond this point, all components of the RYW-TE specimen completely dissolved in the supernatant solution, and the peptide extraction yield decreased for some peptides dissolved in the supernatant.

Furthermore, it can be seen from Figure 1c that the amount of extracted peptide increased upon increasing the rotational speed from 1000 to 3000 rpm, beyond which point the yield decreased once again. These results indicate that RYW-TP was precipitated at a relatively low rotational speed, and precipitation was complete at 3000 rpm. However, at higher rotational speeds, other substances in the supernatant may also precipitate, resulting in a decrease in the relative active peptide content in the specimen.

Moreover, according to the data presented in Figure 1d, the highest extracted peptide yield was achieved after a total of eight extractions, after which the yield decreased once again. Overall, the number of extractions had a relatively small effect on the active peptide content of the extract, and it is likely that upon increasing the number of extractions beyond the optimal value, additional components were also extracted, again leading to a relative decrease in the active peptide content of the RYW-TP specimen.

### 3.2. Optimization of the Peptide Extraction Process

Considering that the above extraction process was the result of multi-factor interactions, it was necessary to further study and evaluate the influence of each factor and its interactions on the active peptide content of the extract. Based on the results of single-factor experiments, three parameters were identified as exhibiting significant effects on the active peptide content, namely the acetone/ethanol ratio, the solid/liquid ratio, and the centrifugal speed, under a fixed number of extractions of eight. The experimental design comprised a combination of three factors and three levels. The Box–Behnken response surface method was used to optimize the extraction process, and 17 experiments were designed with the active peptide content (Y) as the response value. The results are presented in Table 3.

### 3.3. Model Variance Analysis

A one-way ANOVA was used to estimate the significance of the second-order polynomial equation fitting for the experimental data. Response surface regression analysis was conducted using Design Expert 8.0.6 software [20], and the content of active peptides in RYW-TP was used as the response value to obtain the multiple linear regression fitting equation:

Y = 71.00 + 1.88A + 0.40B + 1.52C−0.050AB + 0.45AC + 0.100BC−9.95A^2^ − 1.80B^2^ − 4.15C^2^ (1)

The results of the model significance coefficients and variance analyses are presented in Table 4. The F value of the model was determined to be 229.73 (*p* < 0.0001), and the item misfit was 0.1790 (*p* > 0.05), indicating that the model had a high degree of fit and good reliability. Moreover, the regression coefficient (R^2^) was calculated as 0.9966, thereby confirming that the quadratic regression equation can predict the response value well and that the fit is good. According to the obtained *p* values, the initial acetone/ethanol ratio (A) and centrifugal speed (C) had significant effects on the active peptide content, whereas the amount of solvent (B) had no significant effect. In contrast, for the second combination, A^2^, B^2^, and C^2^ all showed significant effects on the active peptide content, while the interaction terms AB, AC, and BC had no significant effects. According to the F values presented in Table 4, the order of influence of the extraction conditions on the active peptide content was as follows: acetone/ethanol ratio (A) > centrifugal speed (C) > solid/liquid ratio (B).

**Figure 2:**
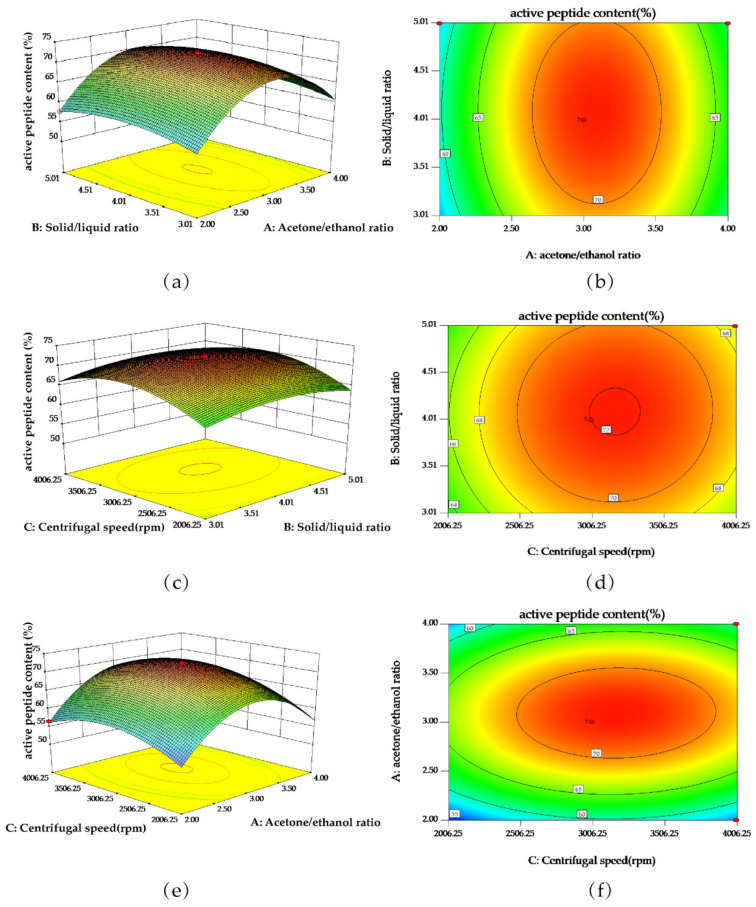
Response surface diagrams showing the effects of various factors’ interactions on the active peptide content: Response surface plots (**a**,**c**,**e**) and the corresponding contour plots (**b**,**d**,**f**) showing the solid/liquid ratio and the acetone/ethanol ratio (**a**,**b**); the solid/liquid ratio and the centrifugal speed (**c**,**d**); the acetone/ethanol ratio and the centrifugal speed (**e**,**f**).

Subsequently, the pairwise interactions between the factors were illustrated using a three-dimensional response surface diagram consisting of the acetone/ethanol ratio (A), the solid/liquid ratio (B), and the centrifugal speed (C), as shown in Figure 2. It can be seen from Figure 2 that the steepest curve was observed between the acetone/ethanol ratio and the centrifugal speed, followed by the solid/liquid ratio and the acetone/ethanol ratio, and the solid/liquid ratio and the centrifugal speed, thereby indicating that the interaction between the solvent proportion (acetone/ethanol) and the centrifugal speed had the most significant influence on the response value, while the other two had a weak influence; this is consistent with the obtained ANOVA results.

Based on the above results, the optimal theoretical conditions were identified as an acetone/ethanol ratio of 3.09:1, a solid/liquid ratio of 4.09:1, and a centrifugal speed of 3190.28 rpm, which gave a predicted active peptide content of 72.33%. For simplicity during practical applications, these conditions were adjusted slightly to give an acetone/ethanol ratio of 3:1, a solid/liquid ratio of four times, and a centrifugal speed of 3200 rpm. Thus, the red millet yellow wine specimen (5 g) was subjected to the optimized extraction process for verification, and the whole process was repeated three times. The average extracted active peptide content was determined to be 72.23%, which is close to the theoretical value, thereby indicating that the designed extraction and separation process was effective, stable, and feasible. These results represent a new understanding of the research and development of polypeptide extraction and separation from red millet yellow wine.

### 3.4. Molecular Weight Distribution of RYW-TP

The determined molecular weight distribution of the RYW-TP sample is listed in Table S1, and the corresponding HPLC results used to calculate these distributions are given in Figure S1. From these data, it can be seen that peptides with molecular weights of <2000 Da accounted for 97.42% of the RYW-TP specimen, revealing that RYW-TP was mainly composed of small molecular active peptides and amino acids.

### 3.5. Analysis of the Amino Acid Composition of RYW-TP

Considering that the amino acid composition, hydrophobicity, and structures have been reported to influence the antioxidant activities of peptides, the amino acid compositions of the peptides present in RYW-TP were evaluated and the results are presented in Table 5 [25]. It can be seen that the RYW-TP contains 17 amino acids that are required by the human body and the total amino acids accounted for 47.22% of the RYW-TP; these findings align with an earlier study on the amino acid profile of Shaoxing rice yellow wine, indicating the feasibility of the method [11]. Furthermore, hydrophobic amino acids accounted for 47.8% of the total amino acids; similarly, Mengjie reported a maximum of 43.6% for this component in Shaoxing yellow wine [11]. These peptides are characterized by high levels of glutamic acid, leucine, aspartate, and alanine. Such hydrophobic amino acids are known to exhibit strong antioxidant capacities due to the exposure of their hydrophobic structures during hydrolysis [26]. These considerations therefore indicate that yellow wine and its peptides should exhibit potential antioxidant properties.

### 3.6. Identification of the Peptide Sequences

The peptides present in the RYW-TP were subsequently identified by Nano-LC-Q-Orbitrap-MS/MS. Peak software 11.5 and de novo sequencing were used to preliminarily analyze the amino acid sequences and molecular weights of the peptides, yielding a total of 326 peptides. According to the millet protein source in the UniProt database, 22 peptides were obtained with −log *p* values > 15, indicating a reliable result.

The specific amino acid sequences and protein sources identified are listed in Table 6, and the corresponding MS/MS results for the 22 peptides are provided in the Supplementary Materials. The BIOPEP, SwePep, and EROP-Moscow databases were used to search for possible peptide sequences [27]. However, no identical peptides were found, thereby indicating that 22 new peptide sequences appeared to be present in the red millet yellow wine specimen.

As indicated, the RYW-TP comprises small peptides with low molecular weights and more hydrophobic amino acids. In the procedure of yeasting, proteins in red millet and other materials were hydrolyzed into different peptides with long or short sequences with the protease produced by fungi, resulting in a rapid increase in peptide concentration. In the subsequent months, the variety and length of peptides may have decreased due to enzymatic hydrolysis to which longer and less charged sequences were more exposed, providing selectivity for nonpolar amino acids during brewing [28,29].

It was found that 14 of the 22 peptide sequences contain highly hydrophobic amino acids at their N termini, wherein the presence of tryptophan (Trp), proline (Pro), valine (Val), and other hydrophobic amino acid residues was expected to enhance the peptide solubility at the lipid/water junction, thereby enhancing the free radical scavenging effect [30]. Moreover, when leucine (Leu) or valine (Val) is present at the N-terminus, the antioxidant activity of the peptide is generally stronger [31]. These considerations indicate that yellow wine and RYW-TP should exhibit potential antioxidant properties.

### 3.7. Cardioprotective Effect of RYW-TP and RYW-TP Against H_2_O_2_-Induced Oxidative Stress in H9c2 Cells

It is well known that the excess production of ROS can cause oxidative damage to the body and lead to a range of diseases. For example, H_2_O_2_, a type of ROS, reacts with the iron present in cells to form highly reactive oxygen radicals. After exposure to H_2_O_2_, H9c2 cells are known to retain their structural integrity, while the antioxidant enzymes present in these cells undergo significant modifications [32]. It was therefore desirable to evaluate the antioxidant capacities of the RYW-TE and RYW-TP samples by measuring the antioxidant enzyme levels in H9c2 cells.

**Figure 3:**
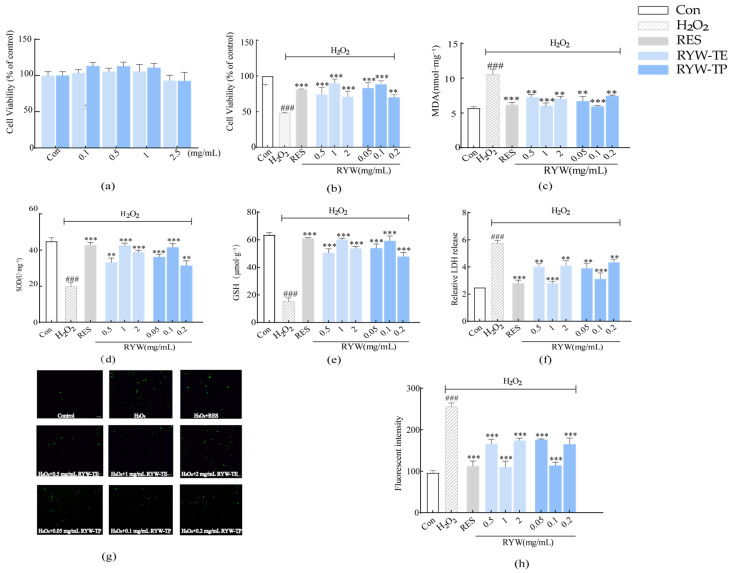
Antioxidant activity of red millet yellow wine and RYW-TP in H9c2 cells. (**a**) Viability of H9c2 cells after exposure to the RYW specimen and red millet yellow wine; (**b**) protective effects of RYW specimen and red millet yellow wine on H_2_O_2_-induced injury in H9c2 cells; variations in the leakage levels of (**c**) MDA, (**d**) SOD, (**e**) GSH, and (**f**) LDH under different treatment conditions; (**g**,**h**) effect of the RYW specimen on the ROS levels in H9c2 cells subjected to H_2_O_2_ damage. Compared with Con group, ### *p* < 0.001; Compared with H_2_O_2_ group, ** *p* < 0.01, *** *p* < 0.001.

As shown in Figure 3a, the viability of normal H9c2 cells was evaluated using the red millet yellow wine samples. Compared with the control group, the cell viabilities of the 2.5 mg/mL RYW-TE and RYW-TP treatment groups were 93.13 and 92.71%, respectively. In addition, the cell viabilities recorded for the 0.1–1 mg/mL treatment groups were >100% (*p* > 0.05). It was therefore apparent that RYW-TE and RYW-TP showed no obvious cytotoxic effects on the H9c2 cells in the concentration range of 0.1–2.5 mg/mL.

To evaluate the protective effects of RYW-TP and RYW-TE on H_2_O_2_-induced H9c2 cell damage, the cells were pretreated with different established concentrations of the sample solutions before exposure to H_2_O_2_ (300 μM), and the results were compared with those obtained using the positive control, resveratrol. As presented in Figure 3b, compared with the H_2_O_2_ group, in which the cell viability decreased to 48.11%, pretreatment with resveratrol (RES, 10 μM), RYW-TE (1 mg/mL), or RYW-TP (0.1 mg/mL) significantly increased the cell viability (*p* < 0.001), giving values of 81.02, 89.74, and 89.47%, respectively. These results indicate that the RYW-TE and RYW-TP specimens exhibited preventive effects against H_2_O_2_ oxidative stress and that these effects were superior to that imparted by resveratrol.

To elucidate the possible protective mechanism of red millet yellow wine on H_2_O_2_-damaged cells, MDA, GSH, and SOD were used to evaluate the levels of oxidative stress, and the LDH leakage rate was employed to evaluate cell damage. In this context, it should be noted that MDA is generated from the action of oxygen-based free radicals on unsaturated fatty acids, and it is often used as a measure of oxidative stress [33]. In addition, SOD, a detoxifying enzyme and the most powerful antioxidant in cells, is known to clear superoxide anions whilst also preventing oxidative damage [34]. Glutathione peroxidase (GSH-Px) and glutathione S-transferase (GST) enzymes are known to remove O^2−^, H_2_O_2_, lipid hydroperoxide (LOOH), and other substances. In addition, GSH maintains the sulfhydryl enzymes in a stable state to prevent oxidation, so it is a key indicator for evaluating the antioxidant ability of a compound. As presented in Figure 3c–f, the MDA and LDH leakage rates in the H_2_O_2_-treated cells were significantly increased, and the levels of GSH and SOD were decreased, indicating that oxidative stress induces myocardial cell injury. However, in the presence of RYW-TE (1 mg/mL) and RYW-TP (0.1 mg/mL), the MDA and LDH leakage rates significantly decreased (*p* < 0.001), whereas the SOD and GSH levels increased (*p* < 0.001). The leakage rates of MDA, SOD, GSH, and LDH were similar to those achieved using resveratrol, indicating that the three components of red millet yellow wine could play preventive roles in relieving H_2_O_2_-induced oxidative stress.

Subsequently, DCFH-DA fluorescent dye was used to evaluate the effects of the RYW components on H_2_O_2_-induced ROS levels in H9c2 cells. As shown in Figure 3g,h, compared with the control group, the fluorescence intensity of the H_2_O_2_ group increased (*p* < 0.001), indicating that the level of ROS increased. After pretreatment with resveratrol (10 μM), RYW-TE, or RYW-TP, the ROS level decreased compared with the H_2_O_2_ group (*p* < 0.001). In addition, it was deduced that RYW-TE and RYW-TP exhibited their most pronounced effects at concentrations of 1 and 0.1 mg/mL, respectively, demonstrating an effect similar to that of resveratrol, and confirming the ROS scavenging effects of red millet yellow wine specimens. These results therefore indicate that red millet yellow wine can alleviate H_2_O_2_-induced oxidative stress damage and improve the antioxidant capacity of cells.

### 3.8. RYW-TE and RYW-TP Upregulated Sirt1/Nrf2 Signaling Pathway in H9c2 Cells

**Figure 4:**
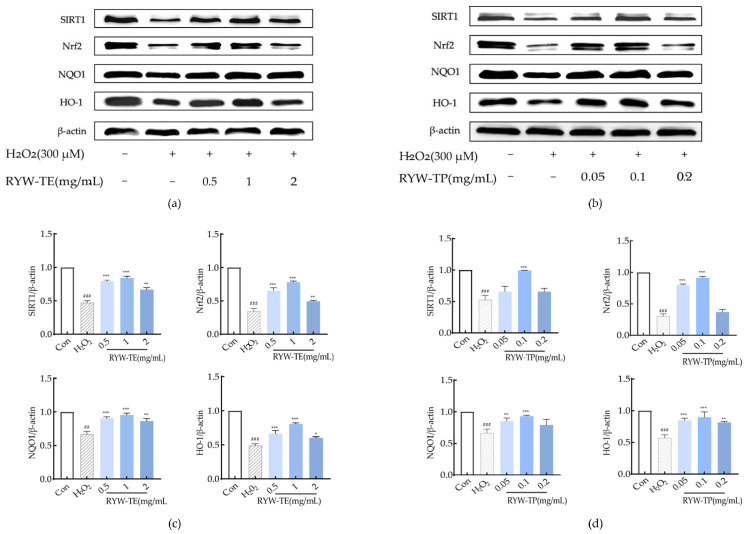
(**a**,**c**) Effects of RYW-TE and RYW-TP treatment on the Sirt1/Nrf2 signaling pathway. (**b**,**d**) Statistical analyses of the expression of (**a**) Sirt1, (**b**) Nrf2, (**c**) NQO1, and (**d**) HO-1 in H9c2 cells. Comparison with the control group, ^##^ *p* < 0.01, ^###^ *p* < 0.001; comparison with the H_2_O_2_ group, * *p* < 0.05, ** *p* < 0.01, and *** *p* < 0.001.

In a previous laboratory study, three kinds of red millet yellow wine and two kinds of glutinous yellow wine were demonstrated to significantly upregulate silent information regulator 1 (Sirt1, an NAD-dependent deacetylase) protein levels in cardiomyocytes, in addition to exhibiting a degree of cardioprotective effect, especially in the case of red millet wheat koji yellow wine [35]. The relevant protective mechanisms were therefore selected for further exploration in the current study. Previous studies have demonstrated that Sirt1 can regulate myocardial oxidative stress and play a role in maintaining cardiac homeostasis [36]. In addition, Lin et al. found that yellow wine polyphenols inhibit oxidative stress by activating the Nrf2 pathway and effectively alleviating doxorubicin (DOX)-induced cardiotoxicity [37]. Thus, Nrf2, an important downstream target gene of Sirt1, was also selected [22,38]. Moreover, considering the increased expression of NQO1 and HO-1 under antioxidative stress, the levels of these species have been widely used as measures of Nrf2 activation [39]. Thus, Sirt1, Nrf2, NQO1, and HO-1 were used to determine whether the peptides present in rice yellow wine can relieve H_2_O_2_-induced oxidative stress by activating the Sirt1/Nrf2 signaling pathway. The results of the Western blotting analyses are shown in Figure 4, wherein it can be seen that in H9c2 cells, the expression of Sirt1, Nrf2, NQO1, and HO-1 was downregulated in the H_2_O_2_ group compared to the control group (*p* < 0.001), whereas protein expression was significantly upregulated upon treatment with RYW-TE and RYW-TP. More specifically, treatment with the optimal RYW-TP concentration of 0.1 mg/mL led to the upregulation of Sirt1, Nrf2, NQO1, and HO-1 expression by 46.53, 60.91, 26.32, and 32.47%, respectively. Similarly, the expression levels of these four proteins increased by 36.94, 43.49, 28.39, and 31.33%, respectively, following treatment with 1 mg/mL RYW-TE, thereby indicating that both RYW-TP and RYW-TE mainly affected the expression of Nrf2. Moreover, the observed increase in Sirt1 expression demonstrates successful activation of the Sirt1 pathway. Since the protein expression of Nrf2, NQO1, and HO-1 was positively correlated with Sirt1 expression, it appeared that Sirt1 upregulated the expression of Nrf2, increased the expression levels of its downstream antioxidant enzymes NQO 1 and HO-1, and enhanced the ability of cells to resist oxidative stress. Collectively, these data indicated that the peptides and red millet rice wine protect cardiomyocytes from antioxidative stress via the Sirt1/Nrf2 pathway.

## 4. Conclusions

In the present study, red millet yellow wine was demonstrated to possess excellent cardioprotective action and bioactive peptides were the main active components responsible for the effect. Such effect was mediated by the antioxidative stress function via upregulation of Sirt1/Nrf2 induced by red millet yellow wine. In addition, a new method was designed to isolate peptides from red millet yellow wine and the maximum peptide yield was up to 72.23%. These results provided the material basis and scientific basis for the cardioprotective effect of red millet yellow wine and demonstrated that drinking red millet yellow wine in moderation is beneficial for health, particularly the prevention of heart disease.

## Figures and Tables

**Table 1:** Single-factor test conditions.

Single-Factor Conditions	Acetone/Ethanol Ratio (X:1)	Solid/Liquid Ratio (Times)	Centrifugal Speed (rpm)	Number of Extractions (Times)
Acetone /ethanol ratio	5, 4, 3, 2, or 1	4	4000	8
Solid /liquid ratio	3	3, 4, 5, or 6	4000	8
Centrifugal speed	3	4	1000, 2000, 3000, 4000, 5000, or 6000	8
Number of extractions (times)	3	4	3000	7, 8, 9, or 10

**Table 2:** Response surface experimental design factors and levels.

Level	Factors		
	(A) Acetone/Ethanol Ratio	(B) Solid/Liquid Ratio	(C) Centrifugal Speed (r/min)
−1	4:1	3	2000
0	3:1	4	3000
1	2:1	5	4000

**Table 3:** Response surface optimization test factors and levels.

Test	(A) Acetone/ Ethanol Ratio	(B) Solid/Liquid Ratio	(C) Centrifugal Speed (rpm)	(Y) Active Peptide Content (%)
1	−1	−1	0	56.8
2	1	−1	0	60.8
3	−1	1	0	57.8
4	1	1	0	61.6
5	−1	0	−1	53.6
6	1	0	−1	56.3
7	−1	0	1	56.6
8	1	0	1	61.1
9	0	−1	−1	63.7
10	0	1	−1	64.2
11	0	−1	1	65.7
12	0	1	1	66.6
13	0	0	0	71.6
14	0	0	0	72.6
15	0	0	0	72.3
16	0	0	0	71.7
17	0	0	0	71.8

**Table 4:** Results of the regression model variance analysis.

Variance Source	Square	Freedom	Mean Square	F Value	*p* Value	Significance
Model	664.57	9	73.84	229.73	<0.0001	**
A	28.13	1	28.13	87.50	0.0001	**
B	1.28	1	1.28	3.98	0.0862	-
C	18.61	1	18.61	57.88	0.0002	**
AB	0.010	1	0.010	0.031	0.8650	-
AC	0.81	1	0.81	2.52	0.1564	-
BC	0.040	1	0.040	0.12	0.7346	-
A^2^	459.80	1	416.85	1430.49	<0.0001	**
B^2^	22.27	1	13.64	69.30	0.0004	**
C^2^	91.04	1	72.52	283.24	<0.0001	**
Residual error	2.25	7	0.35	-	-	-
Missing fit	1.51	3	0.50	2.72	0.1790	-
Net error	0.74	4	0.24	-	-	-
Total deviation	666.82	16	-	-	-	-
R^2^ = 0.9966	R_Adj_^2^ = 0.9923					

Note: **: *p* < 0.01.

**Table 5:** Amino acid composition of the RYW-TP extract (%).

Amino Acid	Abbreviation	Content in RYW-TP (%)
Aspartic acid	Asp (D)	4.24
Threonine	Thr (T)	2.03
Serine	Ser (S)	2.26
Glutamate	Glu (E)	7.04
Glycine	Gly (G)	2.17
Alanine	Ala (A)	3.33
Cystine	Cys (C)	0.31
Valine	Val (V)	2.22
Methionine	Met (M)	2.20
Isoleucine	Ile (I)	3.29
Leucine	Leu (L)	4.54
Tyrosine	Tyr (Y)	2.02
Phenylalanine	Phe (F)	1.73
Histidine	His (H)	1.49
Lysine	Lys (K)	2.58
Arginine	Arg (R)	2.51
Proline	Pro (P)	3.26
Total amino acids	-	47.22
Hydrophobic amino acids	-	22.59

**Table 6:** Peptide sequences in RYW-TP as identified by Nano-LC-Q-Orbitrap-MS/MS.

Amino Acid Sequence	*m*/*z*	Molecular Weight (Da)	Peptide Length	Protein Source
GRGITGPTF	453.2468	904.4766	9	P07728|GLUA1_ORYSJ
VVINPGNPTGQVL	654.3718	1306.7245	13	tr|Q0D4M5|Q0D4M5_ORYSJ
PFKLPPVGP	476.2874	950.5589	9	tr|Q6ZBH2|Q6ZBH2_ORYSJ
DGVLRPGQL	477.7735	953.5294	9	P14323|GLUB1_ORYSJ
KIGGIGTVPVGR	577.3582	1152.6979	12	O64937|EF1A_ORYSJ
PPFNPYENSLNF	719.8403	1437.6565	12	tr|A0A0P0WA63|A0A0P0WA63_ORYSJ
VNPWHNPR	510.2637	1018.5097	8	P14614|GLUB4_ORYSJ
AFRVPTVD	452.7492	903.4814	8	Q6K5G8|G3PC3_ORYSJ
PGIGYPTYPLPR	665.8640	1329.7080	12	Q8H4L8|RA16_ORYSJ
AYGNNIGGYKNE	650.3034	1298.5891	12	P15280|GLGS2_ORYSJ
DQGLGIGSKNPFFNR	825.4290	1648.8322	15	Q84Q83|TOC75_ORYSJ
YVFKHPRPP	380.8831	1139.6239	9	tr|A0A0P0 × 168|A0A0P0X168_ORYSJ
LAFNVPSR	452.2570	902.4974	8	tr|A0A0P0WKY1|A0A0P0WKY1_ORYSJ
VSGAIAGAVSR	494.2842	986.5508	11	tr|Q6Z782|Q6Z782_ORYSJ
LSGTGSAGATIR	545.7982	1089.5778	12	tr|Q33AE4|Q33AE4_ORYSJ
DVIAVPAGVAH	524.7943	1047.5713	11	tr|A1YQH2|A1YQH2_ORYSJ
SGGGGGGGAAHGVL	527.2581	1052.4999	14	Q653V7|AGLU_ORYSJ
LSGTGSVGATIR	559.8130	1117.6091	12	tr|A0A0N7KHX7|A0A0N7KHX7_ORYSJ
SIITTPNPIFSH	663.8585	1325.6979	12	tr|Q65XA1|Q65XA1_ORYSJ
NTGSPITVPVGR	599.3353	1196.6514	12	tr|A0A0P0WQ12|A0A0P0WQ12_ORYSJ
YDIGAGFGH	468.7152	935.4137	9	Q948T6|LGUL_ORYSJ
YTIGGDLGGGEGHNG	702.3138	1402.6113	15	P49027|GBLPA_ORYSJ

## Data Availability

The original contributions presented in the study are included in the article and Supplementary Materials; further inquiries can be directed to the corresponding authors.

## Supplementary Materials

The following supporting information can be downloaded at https://www.mdpi.com/article/10.3390/foods13244111/s1, Table S1: Mw distribution of peptide extracted from RYW-TE; Figure S1. Molecular weight chromatogram of RHJ-TP; Figure S2: The MS/MS figure of the peptides GRGITGPTF; Figure S3: The MS/MS figure of the peptides VVINPGNPTGQVL; Figure S4: The MS/MS figure of the peptides PFKLPPVGP; Figure S5: The MS/MS figure of the peptides DGVLRPGQL; Figure S6: The MS/MS figure of the peptides KIGGIGTVPVGR; Figure S7; The MS/MS figure of the peptides PPFNPYENSLNF; Figure S8: The MS/MS figure of the peptides VNPWHNPR; Figure S9: The MS/MS figure of the peptides AFRVPTVD; Figure S10: The MS/MS figure of the peptides PGIGYPTYPLPR; Figure S11: The MS/MS figure of the peptides AYGNNIGGYKNE; Figure S12: The MS/MS figure of the peptides DQGLGIGSKNPFFNR; Figure S13: The MS/MS figure of the peptides YVFKHPRPP; Figure S14: The MS/MS figure of the peptides LAFNVPSR; Figure S15; The MS/MS figure of the peptides VSGAIAGAVSR: Figure S16: The MS/MS figure of the peptides LSGTGSAGATIR; Figure S17: The MS/MS figure of the peptides DVIAVPAGVAH; Figure S18: The MS/MS figure of the peptides SGGGGGGGAAHGVL; Figure S19: The MS/MS figure of the peptides LSGTGSAGATIR; Figure S20: The MS/MS figure of the peptides SIITTPNPIFSH; Figure S21: The MS/MS figure of the peptides NTGSPITVPVGR; Figure S22: The MS/MS figure of the peptides YDIGAGFGH; Figure S23: The MS/MS figure of the peptide YTIGGDLGGGEGHN.
