# Supplementary material for: Bioactive Peptides and Evaluation of Cardiac Cytoprotective Effects of Red Millet Yellow Wine as Functional Food
Source: Foods. 2024 Dec 19;13(24):4111. doi: 10.3390/foods13244111 (PMC11675123; doi:10.3390/foods13244111)
Supplement: Supplementary file 1 [file foods-13-04111-s001.zip › foods-3363170-supplementary.pdf]

Bioactive Peptides and Evaluation of Cardiac Cytoprotective
Effects of Red Millet Yellow Wine as Functional Food

Zehui He †, Yu Zhou †, Shuang Li, Wen Li, Yingxin Zhang, Cancan Guo, Zexin Guo, Bo Wei * and Yuefeng Bi *

School of Pharmaceutic Sciences, Zhengzhou University, Zhengzhou 450001, China; 18817773761@163.com (Z.H.); zhouyu20001122@163.com (Y.Z.); lis0801@163.com (S.L.)

* Correspondence: weibo@zzu.edu.cn *or* weibozz101@163.com (B.W.); zzubyf@126.com (Y.B.)

† These authors contributed equally to this work.

Table S1. Mw distribution of peptide extracted from RYW-TE

Figure S1. Molecular weight chromatogram of RHJ-TP

Figure S2. The MS/MS figure of the peptides GRGITGPTF

Figure S3. The MS/MS figure of the peptides VVINPGNPTGQVL

Figure S4. The MS/MS figure of the peptides PFKLPPVGP

Figure S5. The MS/MS figure of the peptides DGVLRPGQL

Figure S6. The MS/MS figure of the peptides KIGGIGTVPVGR

Figure S7. The MS/MS figure of the peptides PPFNPYENSLNF

Figure S8. The MS/MS figure of the peptides VNPWHNPR

Figure S9 The MS/MS figure of the peptides AFRVPTVD

Figure S10. The MS/MS figure of the peptides PGIGYPTYPLPR

Figure S11. The MS/MS figure of the peptides AYGNNIGGYKNE

Figure S12. The MS/MS figure of the peptides DQGLGIGSKNPFFNR

Figure S13. The MS/MS figure of the peptides YVFKHPRPP

Figure S14. The MS/MS figure of the peptides LAFNVPSR

Figure S15. The MS/MS figure of the peptides VSGAIAGAVSR

Figure S16. The MS/MS figure of the peptides LSGTGSAGATIR

Figure S17. The MS/MS figure of the peptides DVIAVPAGVAH

Figure S18. The MS/MS figure of the peptides SGGGGGGGAAHGVL

Figure S19. The MS/MS figure of the peptides LSGTGSAGATIR

Figure S20. The MS/MS figure of the peptides SIITTPNPIFSH

Figure S21. The MS/MS figure of the peptides NTGSPITVPVGR

Figure S22. The MS/MS figure of the peptides YDIGAGFGH

Figure S23. The MS/MS figure of the peptide YTIGGDLGGGEGHN

Table S1. Mw distribution of peptide extracted from RYW-TE

Molecular weight range (Da)	Peak area percentage (%, λ = 220 nm)	Numerical mean molecular weight	Weight average molecular weight
>10000	0.02	11012	11080
10000–5000	0.18	6128	6318
5000–3000	0.70	3644	3718
133000–2000	1.68	2358	2389

2000–1000	8.61	1300	1349
1000–500	24.04	650	676
500–180	43.53	271	295
<180	21.24	/	/

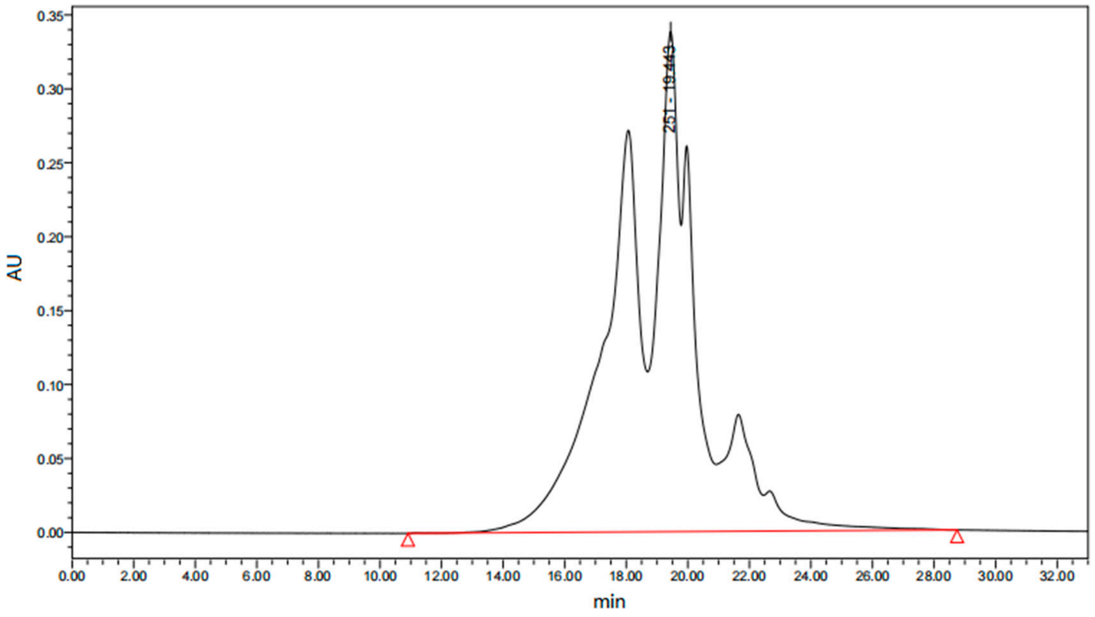

Figure S1. Molecular weight chromatogram of RHJ-TP

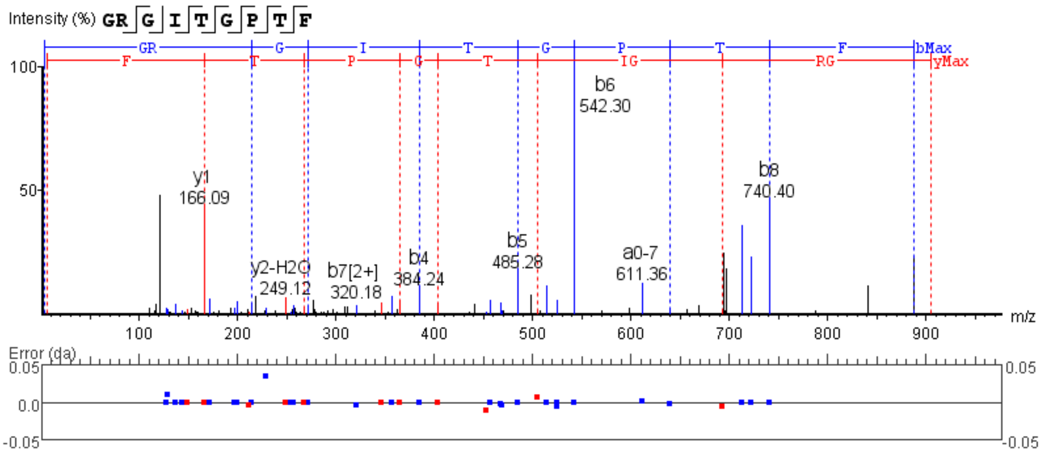

Figure S2. The MS/MS figure of the peptides GRGITGPTF

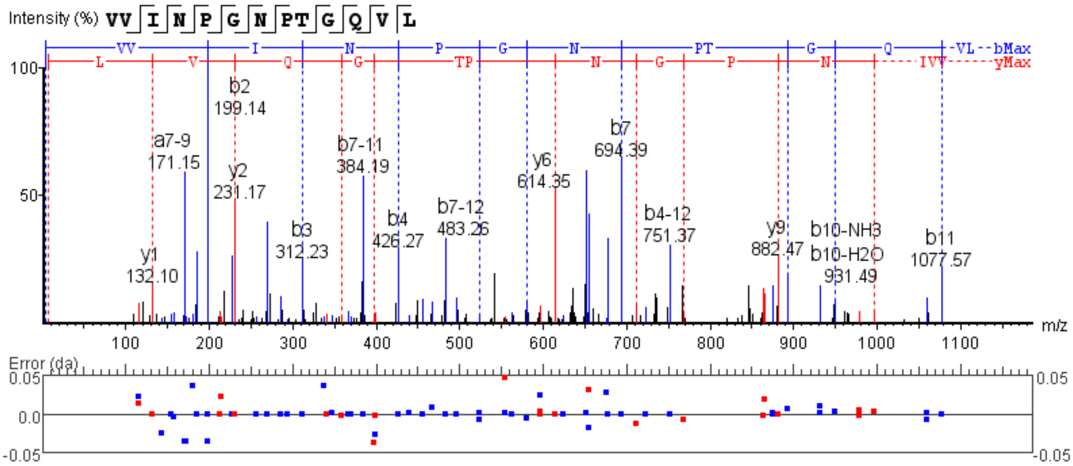

Figure S3. The MS/MS figure of the peptides VVINPGNPTGQVL

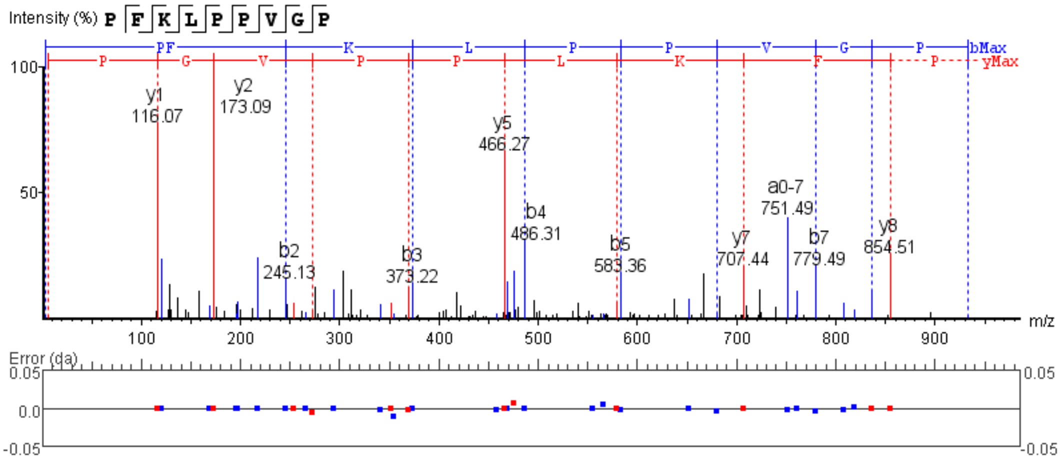

Figure S4. The MS/MS figure of the peptides PFKLPPVGP

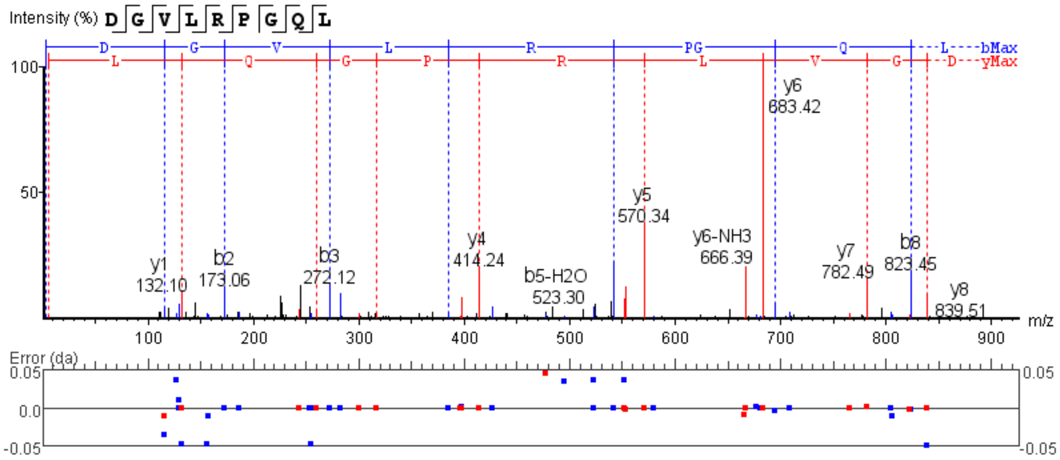

Figure S5. The MS/MS figure of the peptides DGVLRPGQL

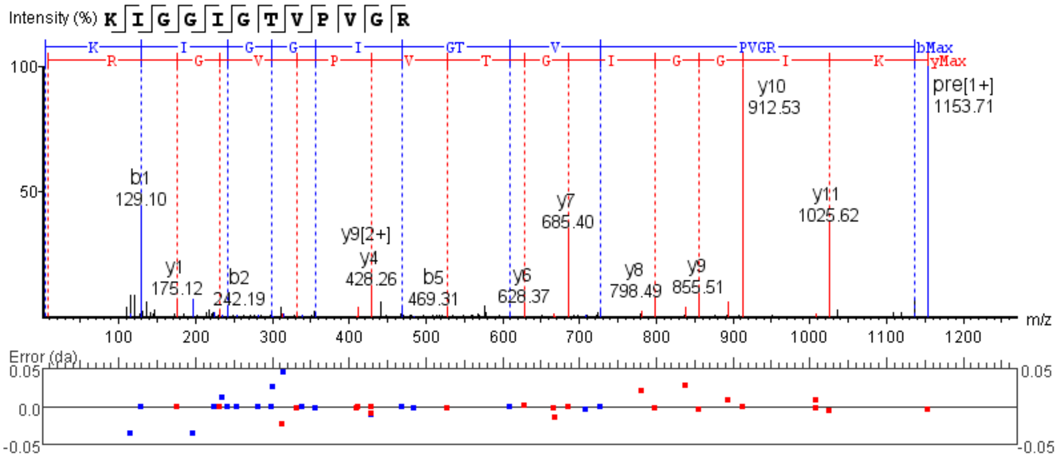

Figure S6. The MS/MS figure of the peptides KIGGIGTVPVGR

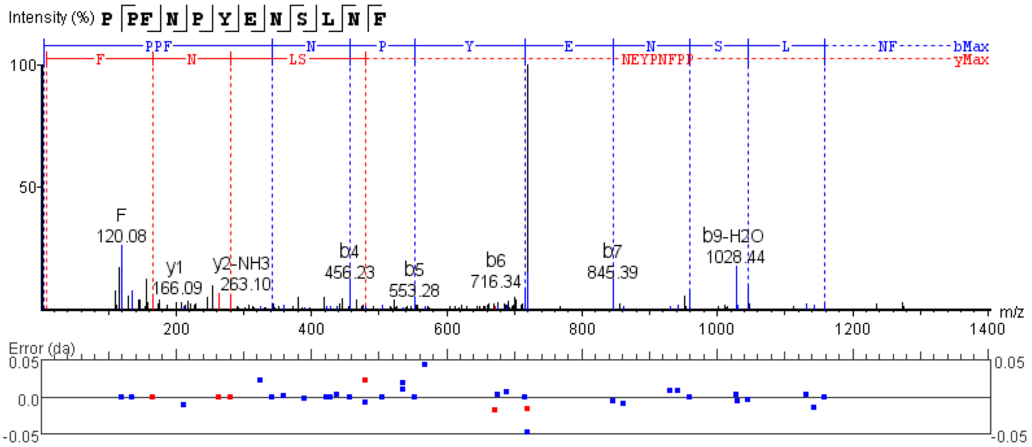

Figure S7. The MS/MS figure of the peptides PPFNPYENSLNF

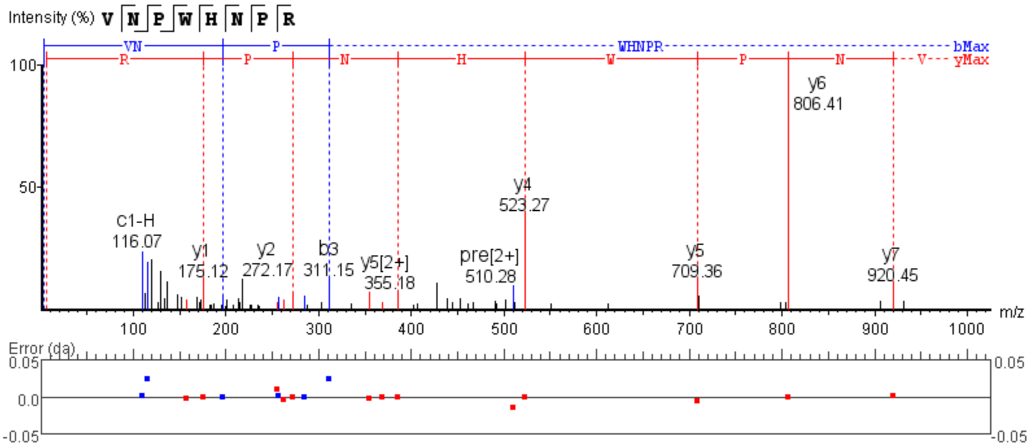

Figure S8. The MS/MS figure of the peptides VNPWHNPR

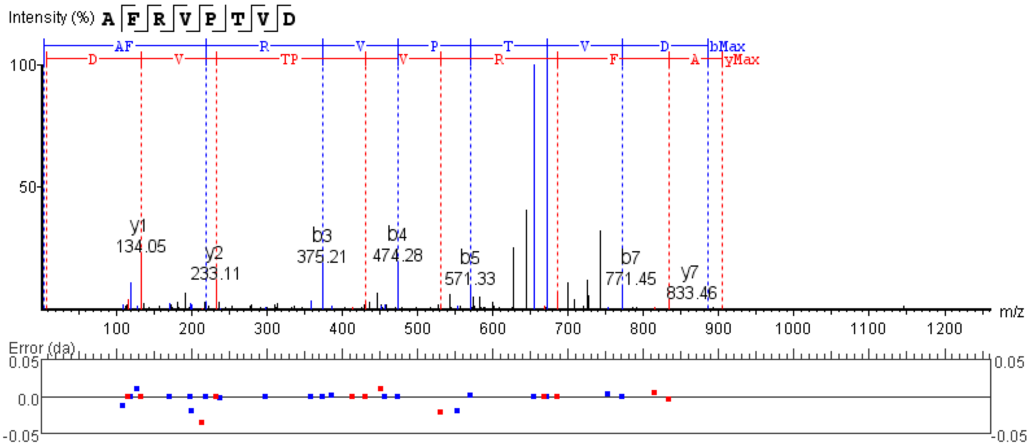

Figure S9. The MS/MS figure of the peptides AFRVPTVD

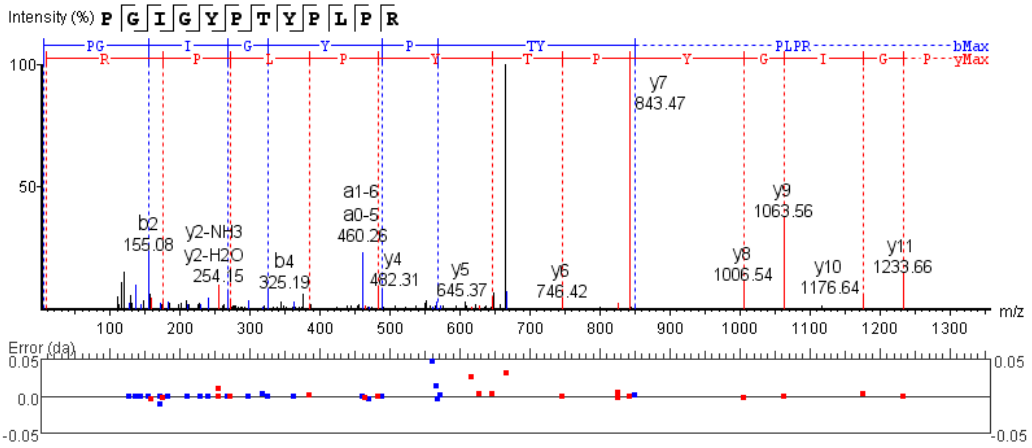

Figure S10. The MS/MS figure of the peptides PGIGYPTYPLPR

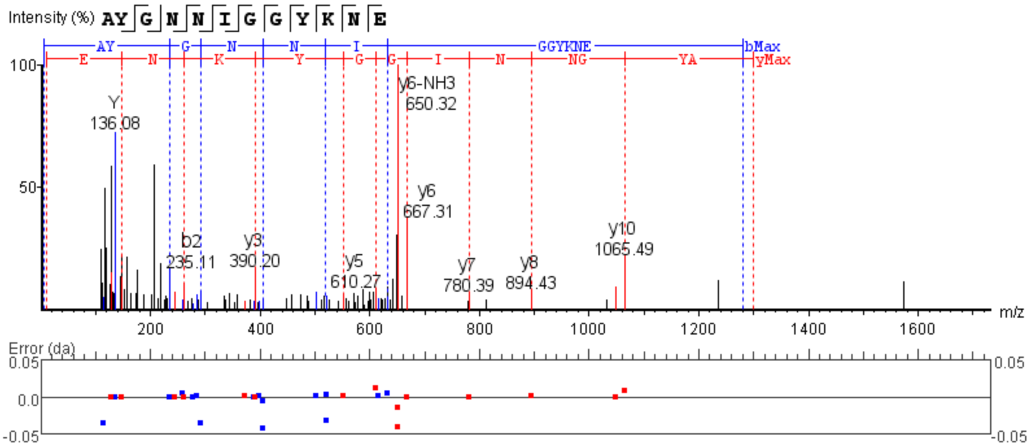

Figure S11. The MS/MS figure of the peptides AYGNNIGGYKNE

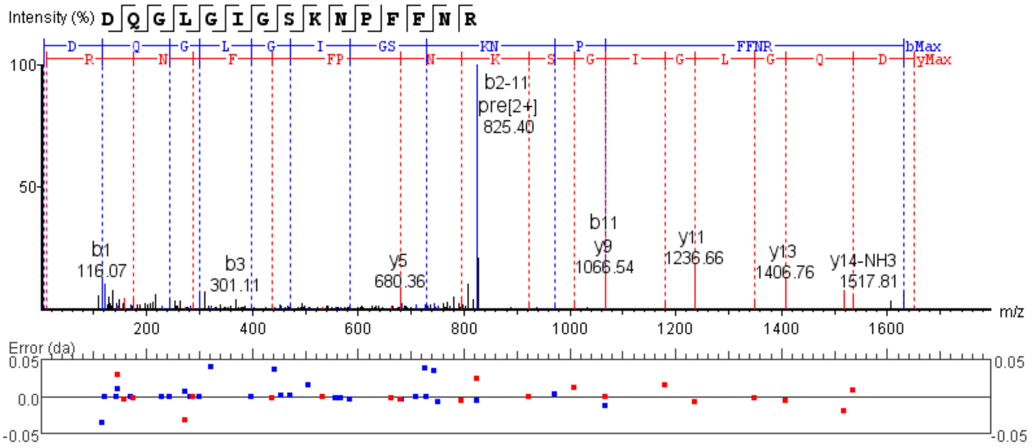

Figure S12 The MS/MS figure of the peptides DQGLGIGSKNPFFNR

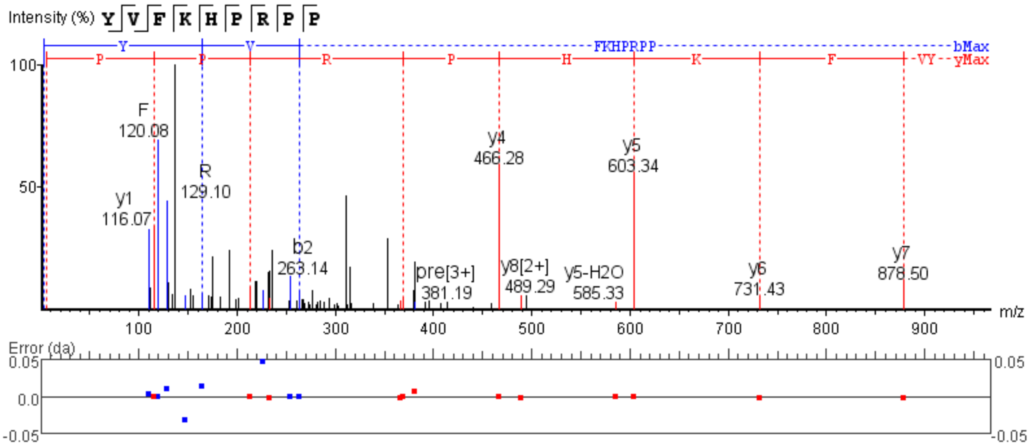

Figure S13. The MS/MS figure of the peptides YVFKHPRPP

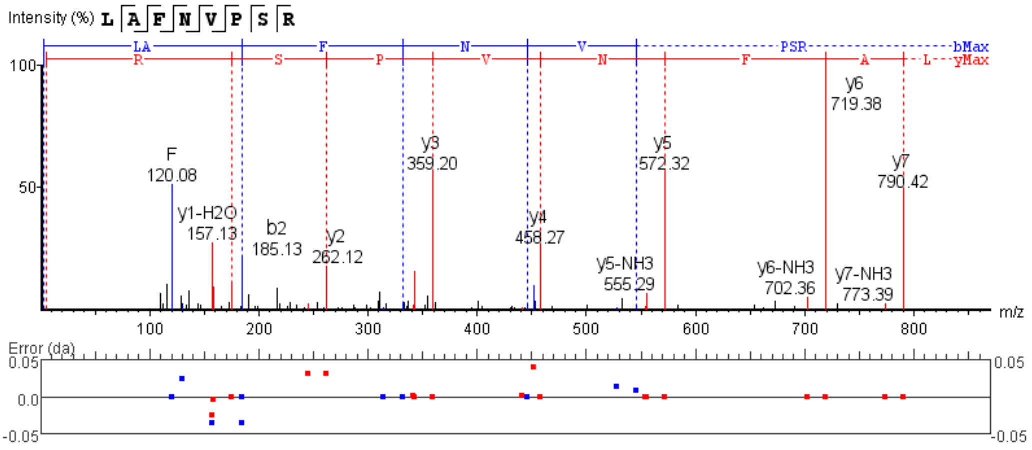

Figure S14. The MS/MS figure of the peptides LAFNVPSR

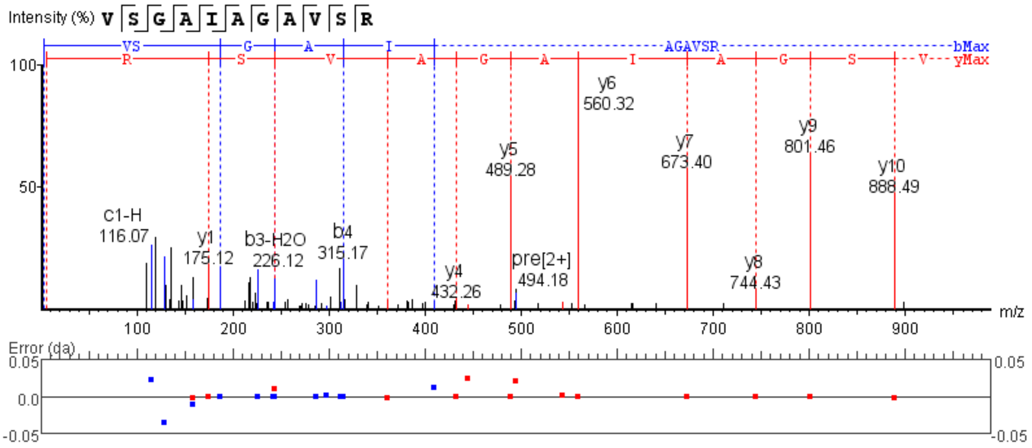

Figure S15. The MS/MS figure of the peptides VSGAIAGAVSR

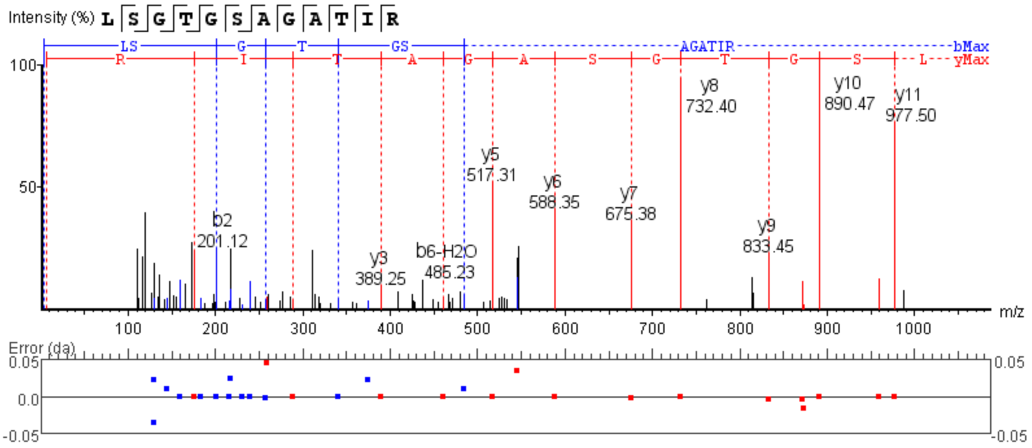

Figure S16. The MS/MS figure of the peptides LSGTGSAGATIR

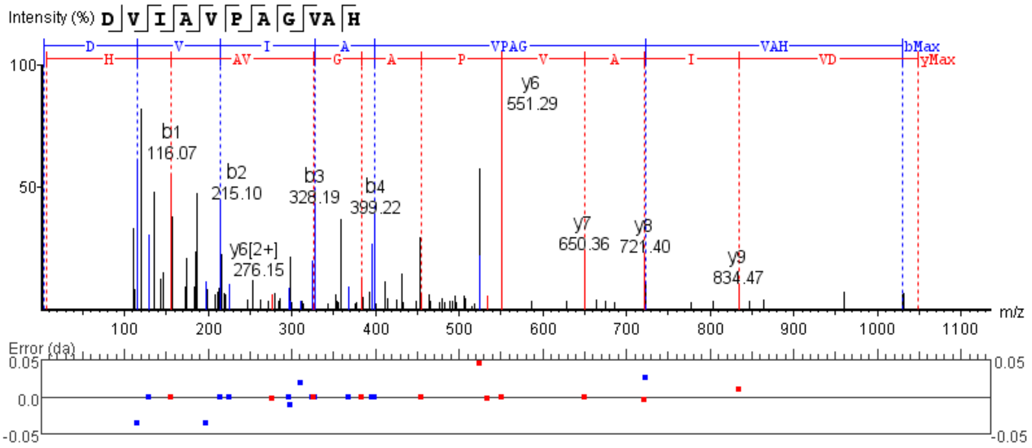

Figure S17. The MS/MS figure of the peptides DVIAVPAGVAH

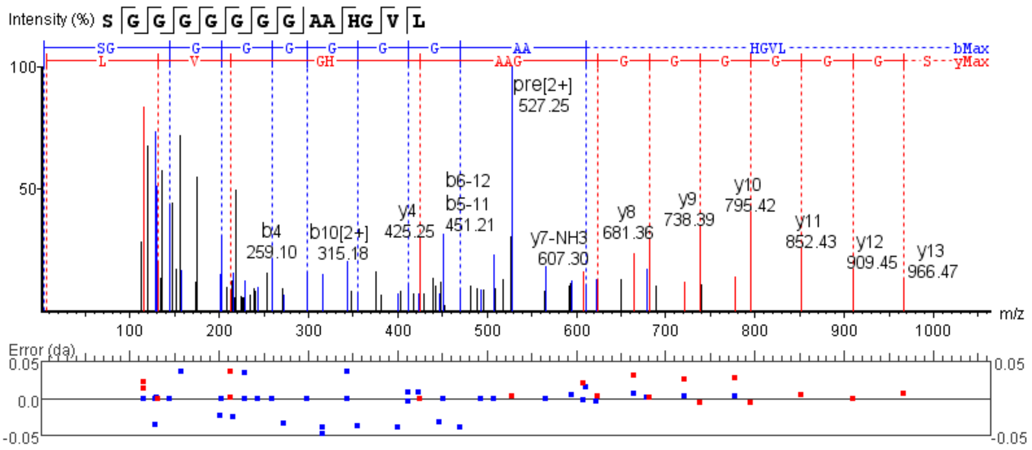

Figure S18. The MS/MS figure of the peptides SGGGGGGGAAHGVL

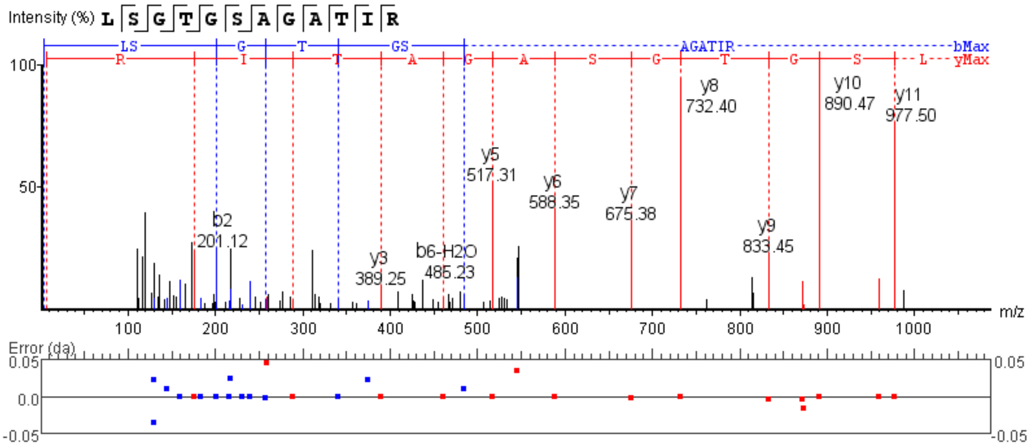

Figure S19. The MS/MS figure of the peptides LSGTGSAGATIR

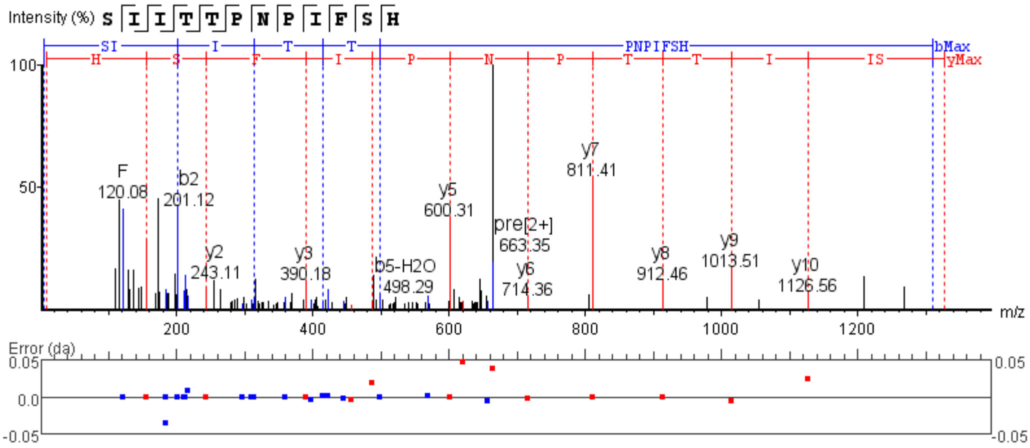

Figure S20. The MS/MS figure of the peptides SIITTPNPIFSH

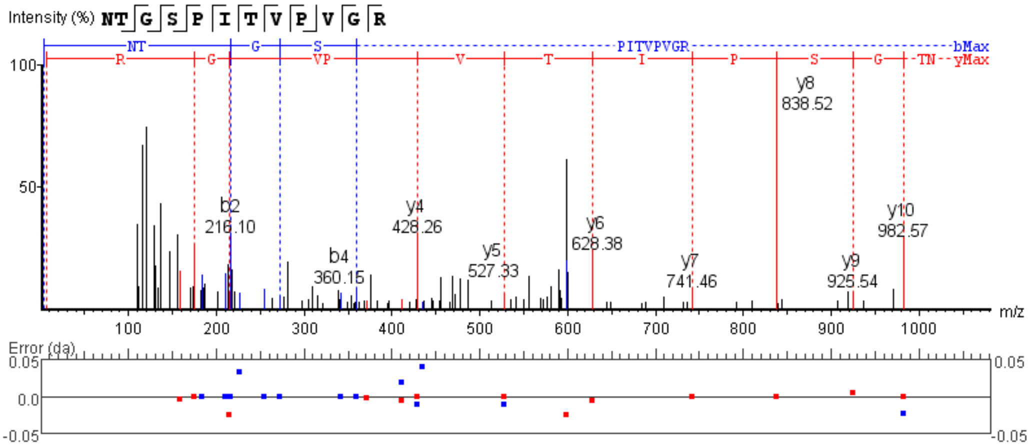

Figure S21. The MS/MS figure of the peptides NTGSPITVPVGR

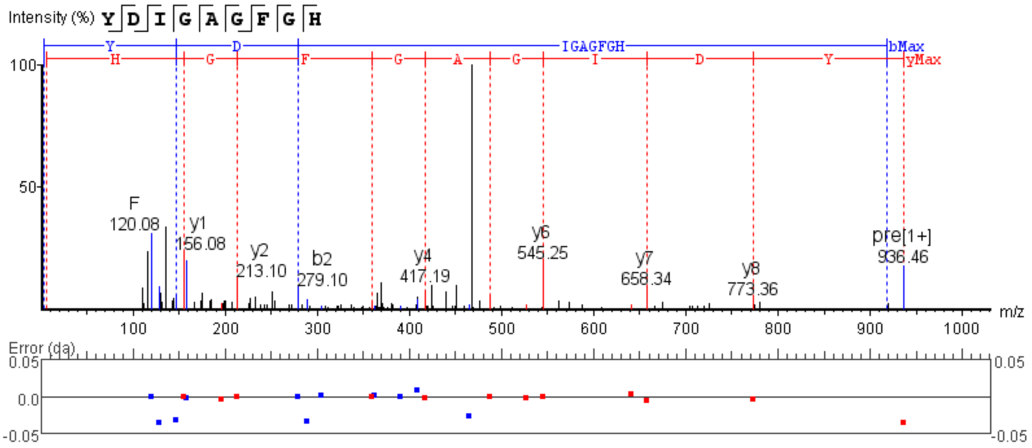

Figure S22. The MS/MS figure of the peptides YDIGAGFGH

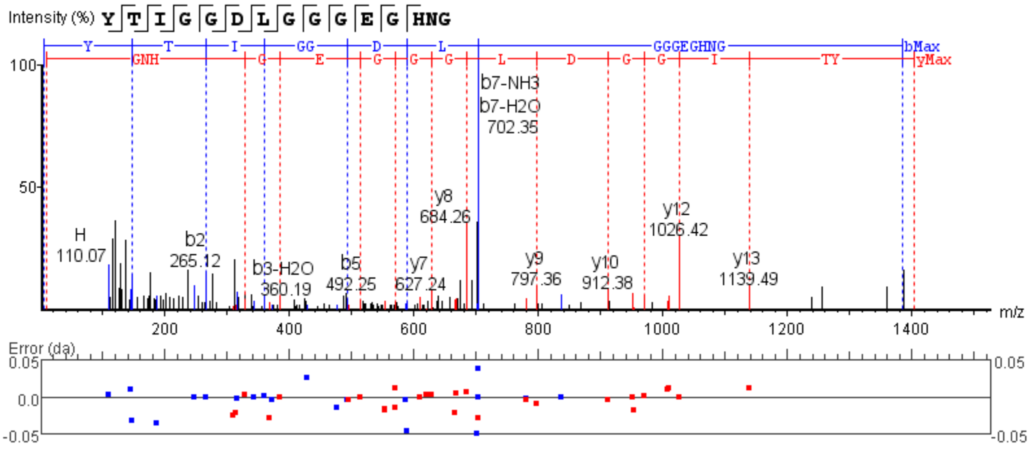

Figure S23. The MS/MS figure of the peptide YTIGGDLGGGEGHN
